# Design and evaluation of a colon cancer mobile application

**DOI:** 10.1186/s12876-024-03275-y

**Published:** 2024-05-28

**Authors:** Amir Sadeghi, Shiva Salar, Pardis Ketabi Moghadam, Makan Cheraghpour, Raziyeh Ghafouri

**Affiliations:** 1 Gastroenterology and Liver Diseases Research Center, Research Institute for Gastroenterology and Liver Diseases, Shahid Beheshti University of Medical Sciences, Tehran, Iran; 2 Student Research Committee, Department of Medical and Surgical Nursing, School of Nursing & Midwifery, Shahid Beheshti University of Medical Sciences, Tehran, Iran; 3 Basic and Molecular Epidemiology of Gastrointestinal Disorders Research Center, Research Institute for Gastroenterology and Liver Diseases, Shahid Beheshti University of Medical Sciences, Tehran, Iran; 4 Department of Medical Surgical Nursing, School of Nursing & Midwifery, Shahid Beheshti University of Medical Sciences, Tehran, Iran

**Keywords:** Cancer, Colorectal Cancer, Mobile Application, Prevention, Screening

## Abstract

**Background:**

Colorectal cancer (CRC) is the third leading cause of cancer and the second cause of cancer-related deaths in the world. Despite the infrastructure and the availability of organized screening programs, participation in their screening programs is less than the set goals. Considering the importance of informing the society about the prevention and early detection of colorectal cancer symptoms and the positive impact of mobile health technologies, the present research was conducted with the aim of designing and evaluating a colon cancer mobile application.

**Methods:**

The present research was conducted in two phases: software design and evaluation. In the first phase, the software was prepared using the cascade method. First, all the educational content related to colorectal cancer was collected through an expert panel with the participation of 10 specialists. Then the software was evaluated with alpha and beta testing, and the initial version was approved by users in terms of simplicity and usability. In the second phase, a parallel clinical randomized trial study was conducted with the aim of investigating the effect of a colon cancer mobile application on the early detection of colorectal cancer. In this stage, 204 volunteers participated; inclusion criteria were age 18–85 years, owning a smartphone and the ability to read and write. Participants were randomized into control and intervention groups. The intervention group was educated with the colon cancer application for education about colorectal cancer, and the control group was educated with a pamphlet. After education, both groups were screened for colorectal cancer symptoms, and the results were compared.

**Results:**

In the software evaluation phase, the application was used by 204 users. In this stage, 84 (41.2%) women and 120 (58.8%) men, with an average (Standard Deviation) age of 47.53 (13.68) participated. Participants were randomized in two groups, 103 people with an average (Standard Deviation) age of 47.62 (14.65) in intervention group and 101 people with an average (Standard Deviation) age of 47.44 (12.70) in control group. There were no significant differences between the demographic characteristics of age, gender, marriage, occupation, instruction level, digestive disease history, cancer history, cancer risk factors, and family history of cancer between the two groups (*P* > 0.05). The Mann-Whitney U test indicated that there is a significant difference between the two groups of participants in self-assessment, willingness to do the screening, and the results of the assessment of colorectal cancer (*P* < 0.05).

**Conclusion:**

The results of the research indicated the positive impact of the Colon Cancer Application on the abilities of the users of self-assessment of colon cancer. Therefore, based on the findings, it can be concluded that the use of the colon cancer mobile application is effective for colon cancer prevention and self-care.

**Trial registration:**

This study was registered in the Iranian Registry of Clinical Trials (https://irct.behdasht.gov.ir) on 13/2/2024, with the IRCT ID: IRCT20210131050189N9.

**Supplementary Information:**

The online version contains supplementary material available at 10.1186/s12876-024-03275-y.

## Introduction

Colorectal cancer (CRC) is the third leading cause of cancer and the second leading cause of cancer-related deaths in the world [1]. In 2020, there were approximately 1.9 million new cases of CRC, which accounted for 935,000 deaths [2]. In Iran, CRC is the fourth most common cancer after stomach, prostate, and lung cancer among men, and among women, it ranks second after breast cancer [3]. The development of CRC results from changes in the healthy epithelium of the colon, including the development of adenomatous polyps that may proliferate, grow, and accumulate genetic and epigenetic mutations over time [4]. Genetic factors, environmental risk factors and lifestyle have significant effects on the increase of CRC. Some of these risk factors include a low-fiber and high-fat diet, a sedentary lifestyle, diabetes, obesity, smoking, alcohol, old age, and inflammatory bowel disease [5].

Although CRC incidence and mortality have declined steadily over the past few decades among adults over 65 years of age, an opposite trend has occurred in adults younger than 50 years, for whom routine screening is not recommended. The alarming increase in young adults combined with the continuing burden of disease in the general population indicates the need to develop new prevention strategies to complement screening [6]. Due to the preventable nature of the disease, the need for public awareness about prevention and early diagnosis with existing screening methods is very important [7]. Early detection is a critical public health strategy in all settings, especially in high-risk populations [8]. CRC is one of the cancers whose mortality can be reduced by 9–32% with regular screening [9].

Given the slow progression of the disease from detectable precancerous lesions and the much better prognosis of patients diagnosed at an early stage, the potential to reduce the disease burden with early detection is significant [4]. To improve the detection and prevention of this disease, finding more accurate, non-invasive and tolerable CRC screening tests has become an urgent need [10]. In cancer prevention and control, the term screening includes the use of evidence-based tests for asymptomatic patients with the aim of identifying early manifestations of neoplastic processes before life-threatening conditions [11]. CRC screening is essential and recommended for all people over 50 years of age [12]. Although, from the point of view of patients, serum testing is better than stool testing, most patients also prefer to avoid colonoscopy, which is very uncomfortable [10]. The effectiveness of screening programs is measured by cancer grade, mortality, survival, and disease incidence (if screening leads to the detection of precancerous lesions, for example, adenomas in CRC), and other factors include acceptability of interventions, quality of life, and affordability is the whole plan [2].

Screening rates vary by age, geographic region, insurance status, race/ethnicity, socioeconomic status, education, and health care resources [13]. Studies in different populations have observed a decrease in CRC incidence and associated mortality with increased screening [14]. Increasing people’s awareness regarding lifestyle modification and recognizing warning signs and symptoms may be effective in reducing complications and mortality from CRC [12]. Despite the infrastructure and availability of organized screening programs, participation in screening programs for many cancers is below the set goals [15].

The use of mobile health technologies (mHealth) in the field of health care is currently widespread and has enabled the rapid sharing of health information [15]. The World Health Organization defines mHealth as the use of mobile or wireless devices for medical and public health activities. There is great potential for mHealth interventions to facilitate healthy lifestyle choices and provide immediate health services [16]. The advantages of using these digital health technologies for cancer prevention are: low cost, high scalability, and providing tailored and specific automatic feedback [11]. MHealth interventions may have a significant impact on participation in cancer screening, especially for breast cancer, cervical cancer, and CRC screening [15].

Various web and internet programs and offline health applications have been designed to improve communication between care providers and recipients and reduce costs [17]. Considering the importance of informing the society about the prevention and early detection of colorectal cancer symptoms and the positive impact of mobile health technologies, the present research was conducted with the aim of designing and evaluating a colon cancer mobile application in two stages: design and evaluation. The purpose of the first stage was to design a colorectal cancer application so that people could study the symptoms and risk factors of colon cancer. The aim of the second stage was to evaluate the application for the early detection of colorectal cancer in its users. The assumption of the research was that application may increase self-awareness about risk factors and symptoms of colorectal cancer and willingness to participate in colorectal screening programs.

## Methods

This study was conducted using the software design method using the cascade model (initial analysis, system analysis, design, programming, testing (alpha and beta), implementation and modification) [18]. In the initial analysis stage, the need or the desired problem, which is the issue of education improvement, is raised. Can technical solutions be provided for it? In the design phase, the application was written by the programmers.

In the next stage (design), the content of the education was prepared using a panel of experts and with the participation of 10 experts from gastroenterology, nursing, nutrition, genetics and pathology, and the initial plan of the program was written. Then the application was prepared for the Android platform. In the next stage (testing stage), the application was tested for errors and weaknesses, and it was checked in terms of user acceptance in two parts, alpha and beta. In the alpha phase, the application was reviewed by the designers themselves as users, and in the beta phase, a group of non-technical users from the research community examined the application and collected their opinions.

At the test phase, the application was examined for errors and weaknesses, and in terms of the user acceptance test, it was examined in two parts, alpha and beta test. In the alpha test, the application is reviewed by the designers themselves as users, and in the beta phase, a group of non-technical users from the community reviews the application [18, 19]. In the alpha test, it was given to 21 specialists (doctors and nurses working in the gastroenterology department and nurses who are members of the academic staff), and they were surveyed about the application. At the beta test, 50 people over the age of 18 were placed, and it was evaluated regarding the applicability, convenience and simplicity of the application.

Then, in the implementation phase, a parallel clinical randomized trial study was conducted with the aim of investigating the effect of using the colon cancer mobile application on the early detection of colorectal cancer. The primary outcome was self-awareness about risk factors and symptoms of colorectal cancer and willingness to participate in colorectal screening programs.

Sampling was conventional. For this stage, we called volunteers for colorectal cancer screening on February 13, 2024; 210 volunteers were enrolled until February 15, 2024, and randomized into two groups. The pretest for colorectal cancer was done on February 16, 2024, until February 18, 2024; intervention was done from February 19, 2024, until March 1, 2024; and the post test was done on March 2, 2024, until March 4, 2024.

The participants were randomly selected from clients of the Ayatollah Taleghani Medical Education Center by a coin toss. Due to the fact that it is important to know and identify the risk factors of cancer at a younger age, such as family members having colon cancer, the age variable was not considered. So, inclusion criteria were age 18–85 years, owning a smartphone and the ability to read and write. Participants were randomized into control and intervention groups. Exclusion criteria was getting flu, COVID, or other illness and do not come for screening of our post. After randomization and education, 2 volunteers in the intervention group and 4 volunteers were excluded from the study because they got the flu.

Randomization of intervention and control groups was done using Study Randomizer [Software Application]. After registering in the software, first the study characteristics, including the number of study groups and the number of participants, were registered in the software. In the next step, a code was assigned to each participant in the order of entering the research, along with a random assignment to the intervention and control groups assigned by the software.

In the intervention group, using the colon cancer software prepared, they were taught about the colon, colon cancer, stages of cancer, its early symptoms, risk factors and ways to reduce the risk of contracting and screening. Their ability to identify early symptoms and self-care were promoted in the prevention of colorectal cancer. Also, there were two self-assessment checklists in the software that users could use to assess their risk of colorectal cancer, and based on each person’s condition, they would be given feedback on what needs further follow-up.

The colorectal cancer self-assessment checklists were prepared by an expert panel of 24 gastroenterology, oncology and nursing specialists. The colorectal cancer risk factors checklist has 12 items (obese, life style, having a history of inflammatory bowel diseases, having a history of polyps and colorectal cancer in the individual and first-degree relatives), and the suspected symptoms of colorectal cancer self-assessment checklist has 4 items (pain during defecation, sudden weight loss of more than 6%, chronic bleeding from the anus, feeling of a lump in the anus). Then its validity was confirmed by the Content Validity Ratio (CVR) and Content Validity Index (CVI), and its reliability was confirmed by Inter-rater Reliability.

In the control group, colon cancer stages, early symptoms, risk factors and ways to reduce the risk of colon cancer and its screening was educated with a pamphlet. After education, both groups were screened for symptoms of colorectal cancer by examining the occurrence of symptoms of abdominal pain during defecation, sudden weight loss of more than 6%, chronic bleeding from the anus, feeling of a lump in the anus, and having a history of inflammatory bowel diseases, having a history of polyps and colorectal cancer in the individual and first-degree relatives.

The study was conducted in a blinded manner. In this way, colorectal cancer self-care education was conducted by researcher A, and the incidence of colorectal cancer symptoms was evaluated by researcher B. Also, the data analyst was blinded the allocation of groups.

### Data analysis

Data analysis was performed using the Statistical Package for The Social Science-20 (SPSS-20). The Kolmogorov-Smirnov test was used to check for the normality of the data. Data analysis included descriptive statistics such as mean, median, interquartile range and standard deviation, as well as inferential statistics such as independent t-test and Mann-Whitney U test. In all tests, a significance level of less than 0.05 was considered statistically significant.

## Results

### First stage (application test)

In the test phase, the written program was checked for errors and weaknesses, and in terms of the user acceptance test, it was checked in two parts, alpha and beta. 5 programming experts with an average (Standard Deviation) age of 37.76 (7.87) and 20 professional nurses and doctors with experience in the gastroenterology department with an average (Standard Deviation) age of 33.8 (7.32) participated in the alpha test. The results of the alpha test are shown in Table 1.

**Table 1:** Alpha test results

	Participants Items	Experts			IT Experts		
		Mean (SD)	Median	IQR	Mean (SD)	Median	IQR
1	Do you think the visual status of the program is appropriate?	4.62 (0.67)	5	1	4.8 (0.44)	5	0.5
2	Do you think the graphic design of the program is suitable?	4.48 (0.75)	5	1	4.4 (0.89)	5	1.5
3	Can the program change the attitude towards the importance of the topic (observing health tips and maintaining health)?	4.62 (0.67)	5	1	4 (1)	4	2
4	Can the program create motivation to observe health tips and maintain health?	4.48 (0.81)	5	1	4.4 (0.89)	5	1.5
5	Are the resources used in the program appropriate?	4.62 (0.67)	5	1	4.6 (0.54)	5	1
Total		22.80 (1.32)	23	2	22.20 (1.48)	22	2.50

50 people over 18 years of age with an average (Standard Deviation) age of 31.20 (11.74) participated in the beta test and commented on the simplicity, practicality and appropriateness of the software. The results of the beta test are shown in Table 2. The results of the test phase indicated that the software is simple and practical, and the content is suitable for the users.

**Table 2:** Beta test results

	Items	Mean (SD)	Median	IQR
**1**	How specialized do you think the contents of the program are?	4.76 (0.48)	5	0
**2**	Do you think the content of the program is enough	4.66 (0.56)	5	1
**3**	Do you think the visual status of the program is appropriate?	4.80 (0.49)	5	0
**4**	Do you think the attractiveness of the program is appropriate?	4.76 (0.56)	5	0
**5**	Do you think the transfer of content is good?	4.68 (0.62)	5	0.25
**6**	Do you think the program is useful?	4.68 (0.65)	5	0
**7**	Do you think it is easy to use the program?	4.64 (0.66)	5	0.25
**8**	Don’t you think the program environment is confusing?	4.76 (0.56)	5	0
**9**	Would you like to recommend the app to others to use?	4.84 (0.47)	5	0
**10**	How do you rate the program?	4.82 (0.48)	5	0
Total		47.46 (1.72)	47.50	3

### The second stage (evaluation)

The present research was conducted with the participation of 84 (41.2%) women and 120 (58.8%) men, with an average (Standard Deviation) age of 47.53 (13.68). In the intervention group, there were 103 people with an average (Standard Deviation) age of 47.62 (14.65) and 101 people with an average (Standard Deviation) age of 47.44 (12.70). The distribution of the age variable was checked using the Kolmogorov–Smirnov test, and it was normal, and its similarity in the two groups was checked using the independent t-test.

Also, the Mann-Whitney U test showed that there were no significant differences between the demographic characteristics of age, gender, marriage, occupation, instruction level, digestive disease history, cancer history, cancer risk factors, and family history of cancer between the two groups (*P* > 0.05) (Table 3).

**Table 3:** Demographic characteristics of the participants

Demographic Characters		Control	App	
		Count (N %)	Count (N %)	Test Result
**Age**	18–39	44 (22%)	42 (21%)	T=-0.09 df = 201 *P* = 0.92
	40–65	51 (25%)	55 (27%)	
	> 65	6 (3%)	5 (2%)	
**Gender**	Female	43 (21.1%)	41 (20.1%)	P_mann_=0.77
	Male	58 (28.4%)	62 (30.4%)	
	Mean Rank	103.69	101.33	
**Marriage**	Single	18 (8.8%)	20 (9.8%)	P_mann_=1.00
	Married	82 (40.2%)	80 (39.2%)	
	Divorced	1 (0.5%)	3 (1.5%)	
	Mean Rank	102.50	102.50	
**Occupation**	High school	63 (33.3%)	75 (39.7%)	P_mann_=0.07
	Undergraduate	19 (10.1%)	13 (6.9%)	
	Graduated	11 (5.8%)	8 (4.2%)	
	Mean Rank	82.44	93.18	
**Instruction level**	Employee	64 (36.8%)	67 (38.5%)	P_mann_=0.12
	Freelance job	27 (15.5%)	15 (8.6%)	
	Unemployed	1 (0.6%)	0	
	Mean Rank	99.94	90.22	
**Having the gastrointestinal disease**	NO	44 (21.6%)	52 (25.5%)	P_mann_=0.33
	YES	57 (27.9%)	51 (25.0%)	
	Mean Rank	106.06	99.00	
**Having the history of cancer**	NO	76 (39.8%)	82 (42.9%)	P_mann_=0.058
	YES	22 (11.5%)	11 (5.8%)	
	Mean Rank	100.94	90.80	
**Having the history of cancer in family**	NO	65 (31.9%)	66 (32.4%)	P_mann_=1.00
	YES	36 (17.6%)	37 (18.1%)	
	Mean Rank	102.36	102.64	
**Screening in the past**	NO	74 (37.4%)	68 (34.3%)	P_mann_=0.52
	YES	26 (13.1%)	30 (15.2%)	
	Mean Rank	97.24	101.81	
**Having the risk factor of colorectal cancer**	NO	57 (27.9%)	68 (33.3%)	P_mann_=0.19
	YES	44 (21.6%)	35 (17.2%)	
	Mean Rank	107.44	97.66	

The Mann-Whitney U test indicated that there is a significant difference between the two groups of participants in identifying suspicious symptoms in self-assessment, willingness to do the next screening, re-doing the self-assessment, and the results of the assessment after the training (*P* < 0.05). (Table 4).

**Table 4:** Comparison of screening results in the form of a self-assessment and after education

		Control	App	Test Result
		Count (N %)	Count (N %)	
**Having suspicious symptoms of colorectal cancer**	NO	72 (35.3%)	58 (28.4%)	P_mann_=0.02
	YES	29 (14.2%)	45 (22.1%)	
	Mean Rank	94.79	110.06	
**Willingness to participate in the next screening**	NO	68 (33.5%)	45 (22.2%)	P_mann_=0.001
	YES	32 (15.8%)	58 (28.6%)	
	Mean Rank	89.48	114.16	
**Screening results after education**	NO	19 (9.3%)	33 (16.2%)	P_mann_=0.03
	YES	82 (40.2%)	70 (34.3%)	
	Mean Rank	109.31	95.82	

## Discussion

The present study was conducted with the aim of designing and evaluating colorectal cancer software. The results of the study indicated the positive effect of the software in identifying suspicious symptoms of colorectal cancer on the participants’ self-evaluation and willingness to participate in screening programs (*P* < 0.05). Elepaño, et al. also, in a systematic review, found that mHealth interventions, such as telephone intervention and text messages, were associated with a 20–46% increase in colorectal cancer screening rates compared to usual care [20]. The results of the current research have shown evidence similar to theirs.

Screening rates vary by age, geographic region, insurance status, race/ethnicity, socioeconomic status, education, and health care resources [13]. Ruco et al. have stated that despite the infrastructure and availability of organized screening programs, participation in screening programs for many cancers is less than the set goals. For example, participation in colorectal cancer screening by stool testing remains below the target of 60% in many Canadian provinces [15]. Shaukat & Levin noted that in the USA, only 67% of patients are up-to-date with CRC screening [21]. Also, Toes-Zoutendijk et al. noted that Participation rates in colorectal cancer screening programs vary, with the highest participation rates in the Netherlands (73.0%) and Basque Country (72.4%), followed by Flanders (54.5%) and France (28.6%) [22].

Therefore, it seems that finding a way to improve the willingness to participate in screening is one of the research priorities in any society. One of the strong points in the results of the present study was that using the application may increase the willingness to participate in screening.

Marzuki et al. also stated that participation in colorectal cancer screening among Malaysians is still low despite the increasing trend in incidence, and more than half of new cases are diagnosed at advanced stages. Improving knowledge through the use of mobile applications may increase participation in screening and thereby increase the chance of disease detection [23]. The results of the present study are evidence of the claim that the use of appropriate mobile applications that provide the possibility of self-assessment for people increases their desire to be screened.

In their study, Marzuki et al. designed the ColorApp mobile application, and after evaluation, they stated that it can be used as a user-friendly tool to educate the community about colorectal cancer [23]. Shaffer et al. also reported similar evidence on the usefulness of providing remote and digital care in their systematic study and emphasized its development [24]. Espina et al. also emphasized the development of mobile applications that can be effective in cancer prevention programs [25]. Based on their research findings, Gautom et al. have suggested the use of smart software and virtual platforms in colorectal cancer screening [26].

With the advancement of communication technology, people today prefer to read through their mobile phones using web browsers or mobile applications compared to traditional printed materials [23]. But the content of the software must be scientific. Ghafouri also emphasizes in his study the up-to-date and preparation of the contents provided in the software under the supervision of experts [17]. Also, in their research, Ketelaers et al. emphasized the preparation of appropriate and specialized content under the supervision of experts in each field [27]. Therefore, it is necessary to update these programs as well as the accuracy and accuracy of their information under the supervision of experts and scientific associations [17]. Based on the results of their meta-analysis study, Elepaño et al. have shown the effectiveness of mHealth interventions in promoting CRC screening, but they also emphasized the existence of certain limitations, including the evaluation of the costs used to set up and maintain the software and prepare it under the supervision of professional experts [25].

In their study, Ketelaers et al. emphasize that a specific approach for the patient can further improve the quality and perceived value of the program, so they suggested that the software that provides patient-centered education is effective in educating the patient [27]. Taking into account the feedbacks that was provided in the self-assessment based on the conditions of each patient in the current research software, it can be said that their results are similar to the current research, and the software that can be used with the data bank can help the patient in care. They are more useful if they guide themselves.

Despite the effectiveness of the detection software, as well as in the desire to screen for colon cancer, it is emphasized that it is scientific, up-to-date and cost-effective to maintain and upgrade them. The next limitation is the emphasis on simple language, the comprehensibility of the content, and its appropriateness to the culture of the software audience. Therefore, it is better to prepare software that is patient-centered and appropriate to the culture of each society, so that it is more acceptable.

## Conclusion

The results of the research indicated the positive impact of the Colon Cancer Application on the abilities of the users of self-assessment of colon cancer. Therefore, based on the findings, it can be concluded that the use of Colon Cancer Application is effective for colon cancer prevention and self-care.

### Limitations

One of the limitations was the provision of software in a national language, while different dialects and languages are common in Iran. Therefore, it is recommended that the next software be in multiple languages or with the possibility of text translation. The next limitation was the inability to install the program on IOS phones, which the owners could not use.

### Electronic supplementary material

Below is the link to the electronic supplementary material.

Supplementary Material 1

## Data Availability

Data is provided within the manuscript or supplementary information files.

## References

[1] Zhu Y, Zhang D-F, Wu H-L, Fu P-Y, Feng L, Zhuang K (2023). Improving bowel preparation for colonoscopy with a smartphone application driven by artificial intelligence. NPJ Digit Med.

[2] Akanbi M, Santiago Rivera OJ, Dutta A, Pratiti R (2023). A review of Community Awareness for Colorectal Cancer Screening and Prevention in North and Central Asian countries. Cureus.

[3] Rahimi F, Rezayatmand R, Shojaeenejad J, Tabesh E, Ravankhah Z, Adibi P (2023). Costs and outcomes of colorectal cancer screening program in Isfahan, Iran. BMC Health Serv Res.

[4] Jayasinghe M, Prathiraja O, Caldera D, Jena R, Coffie-Pierre JA, Silva MS (2023). Colon Cancer Screen Methods: 2023 Update Cureus.

[5] Oruç Z, Kaplan MA (2019). Effect of exercise on colorectal cancer prevention and treatment. World J Gastrointest Oncol.

[6] Song M, Chan AT, Sun J (2020). Influence of the gut Microbiome, Diet, and Environment on Risk of Colorectal Cancer. Gastroenterology.

[7] Olyani S, Ebrahimipour H, Mahdizadeh Taraghdari M, Jamali J, Peyman N (2023). Colorectal Cancer awareness and related factors among adults attending primary Healthcare in North-Eastern of Iran: a cross-sectional study. J Res Health Sci.

[8] Tekiner S, Peker GC, Doğan MC (2021). Colorectal cancer screening behaviors. PeerJ.

[9] Blackman EL, Ragin C, Jones RM (2021). Colorectal Cancer screening prevalence and adherence for the Cancer Prevention Project of Philadelphia (CAP3) participants who Self-identify as Black. Front Oncol.

[10] Gude SS, Veeravalli RS, Vejandla B, Gude SS, Venigalla T, Chintagumpala V (2023). Colorectal Cancer Diagn Methods: Present Future Cureus.

[11] Hesse BW, Kwasnicka D, Ahern DK (2021). Emerging digital technologies in cancer treatment, prevention, and control. Translational Behav Med.

[12] Alzahrani KM, Fallatah SM, Almehmadi RA, Alghamdi JS, Alsulaimani AI, Alkhaldi LM (2022). Colorectal Cancer and its Screening among Public in the Western Region of Saudi Arabia. Cureus.

[13] Jain S, Maque J, Galoosian A, Osuna-Garcia A, May FP (2022). Optimal strategies for Colorectal Cancer Screening. Curr Treat Options Oncol.

[14] Brown JJ, Asumeng CK, Greenwald D, Weissman M, Zauber A, Striplin J (2021). Decreased colorectal cancer incidence and mortality in a diverse urban population with increased colonoscopy screening. BMC Public Health.

[15] Ruco A, Dossa F, Tinmouth J, Llovet D, Jacobson J, Kishibe T (2021). Social media and mHealth Technology for Cancer Screening: systematic review and Meta-analysis. J Med Internet Res.

[16] Janda M, Soyer HP (2020). Using Mobile Health Technology and Social Media for the Prevention and early detection of skin Cancer. Dermatology.

[17] Ghafouri R (2023). Facilitators and barriers of using Mobile for Patient Education. J Patient Saf Qual Improv.

[18] Ali WNAW, Yahaya WAJW, Waterfall -ADDIE, Model. An Integration of Software Development Model and Instructional Systems Design in Developing a Digital Video Learning Application. 2023.

[19] Rodríguez S, Sanz AM, Llano G, Navarro A, Parra-Lara LG, Krystosik AR (2020). Acceptability and usability of a mobile application for management and surveillance of vector-borne diseases in Colombia: an implementation study. PLoS ONE.

[20] Elepaño A, Fusingan AS, Yasay E, Sahagun JA (2021). Mobile health interventions for improving colorectal cancer screening rates: a systematic review and meta-analysis. Asian Pac J cancer Prevention: APJCP.

[21] Shaukat A, Levin TR (2022). Current and future colorectal cancer screening strategies. Nat Reviews Gastroenterol Hepatol.

[22] Toes-Zoutendijk E, Portillo I, Hoeck S, de Brabander I, Perrin P, Dubois C (2020). Participation in faecal immunochemical testing-based colorectal cancer screening programmes in the northwest of Europe. J Med Screen.

[23] Marzuki MFM, Yaacob NA, bin Yaacob NM, Hassan MRA, Ahmad SB (2019). Usable mobile app for community education on colorectal cancer: development process and usability study. JMIR Hum Factors.

[24] Shaffer KM, Turner KL, Siwik C, Gonzalez BD, Upasani R, Glazer JV (2023). Digital health and telehealth in cancer care: a scoping review of reviews. Lancet Digit Health.

[25] Espina C, Feliu A, González Vingut A, Liddle T, Jimenez-Garcia C, Olaya-Caro I (2024). Population-Based Cancer Prevention Education intervention through mHealth: a Randomized Controlled Trial. J Med Syst.

[26] Gautom P, Escaron AL, Garcia J, Thompson JH, Rivelli JS, Ruiz E (2023). Developing patient-refined colorectal cancer screening materials: application of a virtual community engagement approach. BMC Gastroenterol.

[27] Ketelaers SH, Ham Nv P, KAv, Timmers T, Nieuwenhuijzen GA, Rutten HJ (2023). The development and implementation of an interactive application for new ostomy patients. Colorectal Dis.

